# Transactions of the Buffalo Obstetrical Society

**Published:** 1889-02

**Authors:** 


### TRANSACTIONS OF THE BUFFALO OBSTETRICAL SOCIETY.

Stated meeting, Tuesday, November 27, 1888.

The President, DR. R. L. BANTA, in the chair.

DR. A. H. BRIGGS was announced as essayist; subject: "Delayed Second Stage, Due to Retracted Os." Dr. Briggs apologized for not presenting a written paper, on account of an accident to his manuscript.

He commenced his remarks by stating that the proper title of his subject was, "A Hitherto Undescribed Cause of Delay in the Second Stage of Labor."

This subject was suggested to him by observations at the bedside. In the divisions of the stages of labor, he gave the second stage as the period from complete dilatation of the os to final delivery. In anatomical studies of the uterus, it had been observed that its circular structure was composed of two kinds of fibers, viz.: circular and longitudinal. The longitudinal fibers belong to the upper portion of the womb, while in the cervix they were more circular, the two being somewhat interblended.

The phenomena of dilatation consisted in the drawing back of the circular fibers by the longitudinal ones, not alone from the expulsive pressure of the uterine contents, but from contraction of the muscular fibers of the womb, due to the muscular action named. Taking that for granted, one could easily see why dilatation occurs without much pressure of the head. In some cases as the edges grow thinner, they grasp the head like a band or cord, and finally can be felt sliding over it. This produces an agonizing pain, just at the moment the os slips over the head; then there is repose, so far as active contractions of the uterus are concerned, and the patient, too, is quiet.

The speaker called particular attention to the part the longitudinal fibers play at this time. He believed that in many cases of primiparæ, the os grasped the neck just at this period of labor, holding it tightly, which was one cause of delayed second stage.

When watching for the advance of labor at this time, he had been struck with the great delay, notwithstanding active pains, as well as other evidences of apparently progressive labor.

In relating a case where he had met substantially this difficulty, he had gone to fetch his forceps, but, on returning, he found the woman had delivered herself. He had not observed this phenomenon stated in the literature of the subject as a cause of delay in this stage of labor, but was convinced from his observations that it was to be regarded as a factor in, or cause of, delay many times. He commended the subject to the attention of the Society, and invited the views of the members as to the correctness of his observations.

DISCUSSION.

Dr. VanPeyma: I believe this condition to be quite rare, and think that it would be recognized in applying the forceps. I do not remember ever having had a case like those described by Dr. Briggs. Referring to one of the related cases, it would appear that Dr. Pettit had learned not to apply the forceps too early, and perhaps Dr. Briggs's theory suggests a practical explanation of the impropriety of resorting to it too early.

Dr. Dagenais: I have examined Charpentier, and have observed what he says upon this subject. He calls it abnormal contraction of the neck of the womb. I am of the opinion that Dr. Briggs is right in recognizing the condition observed, as, now and then, the cause of delay in the second stage. In case I thought best to apply the forceps in this condition, I should resort to it only under chloroform, which would serve to relax the muscular parts.

Dr. W. D. Greene: I cannot say positively that I have ever seen the condition described by the essayist. In a case where I applied forceps, some years ago, and had them on for an hour, I found, three months later, a rent of the cervix, and this was possibly one of the sort of cases described by Dr. Briggs.

Dr. Lothrop: Though this subject is one of interest, I have, I confess, never detected it; but I can well understand how, under the old régime, ergotism might be a potential cause of this contraction of the os around the neck of the child, and that it might have been a more frequent anomaly than now. So, too, I can understand how occasionally in primiparæ, who have marked nervous temperaments, it might possibly occasionally occur. In regard to the forceps, frequent mention having been made of that instrument, it should never be applied without full dilatation, and this I especially insist upon. A study of the causes, anatomical and physiological, that lead to dilatation of the cervix, are interesting both theoretically and practically. Hodge considers dilatation a vital phenomenon, and that the bag of waters has nothing whatever to do with it. Though I have never detected, as I have before said, the condition under discussion, I have yet experienced difficulty often in delivering the shoulders after the head has been born. I have attributed this to large shoulders, but, in exceptional instances, it may have been due to the condition named by the essayist. There is an extreme possibility of its occurrence, but, in my opinion, an extreme improbability of its happening.

Dr. Hartwig: I have no definite opinion as to the frequency with which this condition may occur, but I have no doubt that it does, now and then, happen. Considering the theory of the observation of Dr. Briggs, I would refer to its parallel in hour-glass contractions, and I desire to point out, as showing the power of muscular contractibility, that it is often difficult to pass the hand into the womb, even under chloroform narcosis. I recall a case where I expected much difficulty in the manipulations necessary to obtain a forceps delivery, but in reality I found it very easy of execution, and the child was small. This was probably such a case as the paper refers to.

Dr. Fell commented upon the physiology of dilatation, and believed there were other factors in bringing it about besides the pressure of the extruding forces.

The President: I desire to say a word of compliment to the author, and his method of presenting the subject. Charpentier says that this contraction usually occurs at the internal os. The contraction referred to has been mentioned as usually occurring in primiparæ; why may it not also happen with multiparæ? For, sometimes in the latter cases there is slow dilatation of the parts, or perhaps a deformed head, and when it finally becomes moulded, its slipping into the pelvic cavity is very slow work. I think that the perineum is not often torn by the shoulders, as has been claimed by some observers, and referred to in the discussion. If chloroform is given at this time, it is my opinion that it will release the contraction described by the essayist.

Dr. Briggs, in conclusion: I presented the subject in this form rather than disappoint the Society, and thus stood in the relation of a stop-gap, but realizing that it was imperfectly done. The position I have taken is, I confess, somewhat theoretical, but my observations have led me to think that contraction of the os, as described, affords a possible explanation of delay in the second stage—a delay we have all of us, no doubt, clinically observed. I have seen a case in which the forceps could be applied only with the greatest difficulty, yet the head was small and the pelvis roomy. What else could have retarded labor, excepting a contracted os uteri, grasping and encircling the child's neck? A case, in practice, I have seen like the following: The woman advanced in years, with plenty of pelvic room; I applied the forceps after waiting some time, then chloroform was administered, and in an instant the child was born. This would seem to illustrate a particular reason for the use of chloroform. I wish to say that in the cases I have referred to, ergot had not been used. I believe the treatment, for the condition described, is simply the administration of an anesthetic.

Dr. VanPeyma, by permission, the debate having closed: I desire to call attention to the administration of ergot in what I may term tonic doses. My attention was called to this method of using this drug by Dr. Stockton, and I believe it will oftentimes prove serviceable when there is delay from uterine inertia, in cases that are otherwise normal. Ergot is to be administered in these cases in doses of four or five minims, every twenty minutes, until four or five doses have been given. I have observed that within two or three hours normal pains begin, and very soon delivery is terminated; whereas, in similar cases, without this method, I have waited twenty-four hours.

